# Modeling the Effect of Hypoxia on Macrobenthos Production in the Lower Rappahannock River, Chesapeake Bay, USA

**DOI:** 10.1371/journal.pone.0084140

**Published:** 2013-12-31

**Authors:** Samuel Kersey Sturdivant, Mark J. Brush, Robert J. Diaz

**Affiliations:** 1 Division of Marine Science and Conservation, Nicholas School of the Environment, Duke University, Beaufort, North Carolina, United States of America; 2 Virginia Institute of Marine Science, College of William & Mary, Gloucester Point, Virginia, United States of America; Dauphin Island Sea Lab; University of South Alabama, United States of America

## Abstract

Hypoxia in Chesapeake Bay has substantially increased in recent decades, with detrimental effects on macrobenthic production; the production of these fauna link energy transfer from primary consumers to epibenthic and demersal predators. As such, the development of accurate predictive models that determine the impact of hypoxia on macrobenthic production is important. A continuous-time, biomass-based model was developed for the lower Rappahannock River, a Bay tributary prone to seasonal hypoxia. Phytoplankton, zooplankton, and macrobenthic state variables were modeled, with a focus on quantitatively constraining the effect of hypoxia on macrobenthic biomass. This was accomplished through regression with *Z'*: a sigmoidal function between macrobenthic biomass and dissolved oxygen concentration, derived using macrobenthic data collected from the Rappahannock River during the summers of 2007 and 2008, and applied to compute hypoxia-induced mortality as a rate process. The model was verified using independent monitoring data collected by the Chesapeake Bay Program. Simulations showed that macrobenthic biomass was strongly linked to dissolved oxygen concentrations, with fluctuations in biomass related to the duration and severity of hypoxia. Our model demonstrated that hypoxia negatively affected macrobenthic biomass, as longer durations of hypoxia and greater hypoxic severity resulted in an increasing loss in biomass. This exercise represents an important contribution to modeling anthropogenically impacted coastal ecosystems, by providing an empirically constrained relationship between hypoxia and macrobenthic biomass, and applying that empirical relationship in a mechanistic model to quantify the effect of the severity, duration, and frequency of hypoxia on benthic biomass dynamics.

## Introduction

Macrobenthic organisms (retained on a sieve size >500 µm) are of importance to ecological processes in estuarine ecosystems like Chesapeake Bay [1], regulating or modifying most physical, biological, chemical, and geological processes [2]. Macrobenthos influence these sediment geochemical and physical properties [3] through bioturbation, the biological reworking of sediments [4]. In the estuarine environment, macrobenthos are a primary pathway through which organic carbon is cycled in the sediments [1]. However, the role of macrobenthos is limited to normoxic conditions; anaerobic metabolism becomes an increasingly important means of recycling organic carbon during hypoxia [5]. Macrobenthos also serve as a major energetic link between primary producers and demersal fish and epibenthic predators [6]; however, the sessile nature of macrobenthos makes them susceptible to natural and anthropogenic perturbations such as hypoxia [7], a significant concern given the documented importance of estuarine macrobenthic communities [1].

Since colonial times, the number of humans in Chesapeake Bay watershed has grown exponentially, with a 3-fold increase during the last 100 years [8]. Human activity adversely affects land topography, chemistry of the Earth's atmosphere and water, rates and balance of biogeochemical processes, and biodiversity [9]; Chesapeake Bay estuary is no different. Anthropogenic disturbance has greatly increased the flux of nitrogen and phosphorous compounds through land clearing, application of fertilizer, discharge of human waste, animal production, and combustion of fossil fuels, leading to eutrophication of the Bay [10]. Hypoxia, dissolved oxygen (DO) concentrations ≤2 mg O_2_ l^−1^ [11], is closely associated with eutrophication, an increase in the rate of supply of organic matter to a system [12]. Low DO concentrations have been documented in mainstem Chesapeake Bay since the early 1930s [13] and in the Potomac since the 1910s [14]. Presently, seasonal hypoxia forms in early to late spring and lasts approximately 120 days, with the most severe low DO events occurring during mid-summer in mainstem Chesapeake Bay [8]. From the 1950s to the present, hypoxic volume in the Bay has increased substantially, from approximately 3 km^3^ to 10 km^3^ [15]. This increase is of concern given documentation of low DO impairing growth and reproduction and stressing living resources, increasing faunal susceptibility to disease and other environmental stressors [11], [16]–[25].

As hypoxia continues to increase in the Bay and many other coastal systems worldwide [15], [26], the development of accurate predictive models that quantify the ecological impacts of hypoxia becoming increasingly important. Recent models have begun to include functions relating hypoxia to increased mortality and/or reduced filtration or ingestion of macrobenthos, fish, and shellfish (e.g. [27]–[30]), but typically these functions are hypothetical and have yet to be constrained by empirical data. Models generally take two forms; detailed complex models that attempt to replicate as much of the natural environment as possible [28], [31], and more simplistic models that only incorporate what is functionally necessary [32]. The focus of the current study was to model the effect of observed hypoxia, and various scenarios of hypoxia, on the biomass of macrobenthos; therefore our approach was to force DO concentrations into a simple model to predict macrobenthic responses, rather than to first simulate hypoxia with a highly resolved, linked hydrodynamic-water quality model. The approach we developed here can be incorporated into more complex models as they continue to add additional mechanistic detail with respect to ecological responses to hypoxia. We began by empirically constraining a formulation for hypoxia-induced mortality, incorporated this function into a simplified version of the Chesapeake Bay Eutrophication Model [28], and used the resulting model to predict the potential effects of hypoxia on macrobenthos in the Rappahannock River, a tributary of Chesapeake Bay that experiences seasonal hypoxia.

## Materials and Methods

### 2.1 Study Area

Seasonal hypoxia occurs throughout Chesapeake Bay and some of its tributaries during the summer months [8], but in the lower Chesapeake Bay, the Rappahannock River is the only major tributary with the hydrography that allows for the development of sustained seasonal hypoxia [33]. In the Rappahannock, a combination of tidal mixing and advection of undersaturated mainstem waters into the tributary controls the seasonal hypoxia, which develops in late May and abates in early September [34], [35]. Macrobenthic taxa in the Rappahannock River exhibit a variety of life history traits, such as resiliency and recruitment, which affects their response to seasonal hypoxia [1]. These responses allow some taxa to rapidly recruit to areas post-hypoxia, while other species struggle to re-establish, influencing variation in macrobenthic biomass; information on the functional characteristics of the dominant macrobenthos taxa in the lower Rappahannock are displayed in Table 1. No permits were required for the described study, which complied with all relevant regulations.

**Table 1:** The major macrobenthic taxa of the lower Rappahannock River.

Taxon	Feeding Type	Mobility	Sediment Reworking Rate (mg dry wt. of indiv)	Hypoxia LT_50_ (h)	Reproduction	Notes	Source(s)
*Acteocina canaliculata* (G)	C	M	2080 (displacement)				Myers 1977; Schaffner 1987
*Capitella spp*. (A)	HD	LM		168–312	Spawn (throughout the year) Larval development completely benthonic (∼1 mo.)		Rosenberg 1972; Warren 1976; Kravitz 1983
*Heteromastus filiformis* (A)	HD	LM	262.5–700	312	Spawn (Spring)	Eggs deposited in the sediment wrapped in a cocoon. Hatched larvae may start their burrowing mode of life immediately.	Rosenberg 1972; Cadee 1979; Kravitz 1983; Hines and Comtois 1985
*Mediomastus ambiseta* (A)	HD	LM	262.5–700		Spawn (Apr-Sept)		Kravitz 1983; Schaffner 1987
*Nereis succinea* (A)	SD,O	LM	3.9–103.7	62–84	Spawn (Mar-Oct)	Planktonic larvae remain in the water column until they possess 9–12 segments, at which time they settle to the benthos	Cammen et al. 1978; Fauchald and Jumars 1979; Kravitz 1983; Hines and Comtois 1985; Hardedge et al. 1990; Fong 1991; Sagasti etal. 2001; Tiffany et al. 2002
*Paraprionospio pinnata* (A)	SD,SF	LM	39.6–51.9 (juveniles) 90.9–232.8 (adults)		Spawn (summer) Recruitment (June-Dec) Clutch Size (6000 eggs)	Dominant during hypoxia	Dauer et al. 1981; Kravitz 1983; Schaffner 1987; Luckenbach et al. 1988,;Mayfield 1988
*Streblospio benedicti* (A)	SD,SF	LM	11.7–18.7	27–43	Spawn (spring and summer) Clutch Size (15–70 planktotrophy or 70–460 lecithotrophy)	Larvae to Juvenile (3 days – 2 weeks)4 annual cohorts	Dauer et al. 1981; Hines and Comtois 1985; Kravitz 1983; Levin and Hugget 1990; Llansó 1991; Levin and Bridges 1994
*Tubificoides spp.* (A)	HD	LM	20.7–31.9	720			Tevesz et al. 1980; Hines and Comtois 1985; Giere et al. 1999

Compilation of the functional characteristics of major macrobenthos taxa in the lower Rappahannock River. Feeding type: C- carnivore, HD- head-down, SD- surface deposit, SF- suspension feeder, O- omnivore. Mobility: M- mobile, LM- limited mobility.

### 2.2 Field Collection

Macrobenthos data from a previous study were used to develop a formulation for hypoxia-induced mortality in our benthic model [36]. During the summers of 2007 and 2008, two random sites were chosen in each year in the lower Rappahannock for continuous monitoring of water quality, and biweekly sampling of macrobenthos from May to October. Based on water quality data from the Chesapeake Bay Long-Term Benthic Monitoring Program (www.baybenthos.versar.com) one normoxic site and one site known to experience seasonal hypoxia were chosen in each year. Data from a hypoxic and normoxic site in both 2007 and 2008, four sites total, were used for model construction.

At each monitored location a Hach DS500X Hydrolab datasonde was deployed approximately 0.5 m above the sediment surface attached to a small tripod. DO concentration, salinity, temperature, and depth were recorded every 20 minutes. Approximately every two weeks the datasonde was replaced with another Hydrolab datasonde, and a sediment sample was collected with a Young grab (440 cm^2^ to a depth of 10 cm) for benthic community analysis. Grabs were sieved in the field through a 0.5 mm screen, and organisms and detritus retained on the screen transferred into labeled jars, preserved in a 10% formaldehyde solution, and stained with Rose Bengal. Samples were processed to identify and enumerate each species present as described in Dauer and Llansó [37]. Ash-free dry weight (AFDW) biomass was measured for each species by drying to a constant weight at 60°C and ashing in a muffle furnace at 500°C for four hours.

### 2.3 Model Construction

A continuous-time, biomass-based model was constructed using STELLA Modeling and Simulation Software®. The model was based on the benthic sub-model in the 2002 Chesapeake Bay Eutrophication Model [28], [38], and contained three governing equations. All formulations and parameter values from the parent model [28], [38] were used in the current project with the exception of the modifications described in sections 2.3, 2.4 and 2.5. Phytoplankton biomass was modeled as:
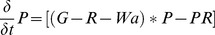
(1)where:

*P*  =  phytoplankton biomass (g C m^−3^)

*G*  =  growth rate of phytoplankton (d^−1^)

*R*  =  respiration rate of phytoplankton (d^−1^)

*Wa*  =  phytoplankton settling loss (d^−1^)

*PR*  =  predation on phytoplankton (g C m^−3^ d^−1^).

Zooplankton was modeled as the combined biomass of micro- and mesozooplankton for simplicity as:
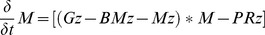
(2)where:*M*  =  zooplankton biomass (g C m^−3^)

*Gz*  =  growth rate of zooplankton (d^−1^)

*BMz*  =  basal metabolic rate of zooplankton (d^−1^)

*Mz*  =  hypoxic mortality (d^−1^)

*PRz*  =  predation on zooplankton (g C m^−3^ d^−1^).

Macrobenthos were modeled as the combined biomass of deposit and suspension feeders, as:
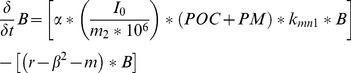
(3)where:

*B*  =  macrobenthos biomass (g C m^−2^)

*α*  =  assimilation efficiency for carbon*I_0_*  =  ingestion rate of macrobenthos (g sediment g C^−1^ biomass^−1^ d^−1^)

*m_2_*  =  sediment solids concentration (kg l^−1^)*POC*  =  sediment particulate organic carbon concentration (g C m^−3^)

*PM*  =  phytoplankton and zooplankton biomass (g C m^−3^)*k_mn1_*  =  Michaelis-Menton growth limitation term for carbon*r*  =  respiration rate of macrobenthos (d^−1^)

*β*  =  predation rate (m^2^ g C d^−1^)

*m*  =  hypoxia mortality rate (d^−1^).

Phytoplankton and zooplankton groups were included in the model given the tight benthic-pelagic (B-P) coupling that exists in estuarine and shallow coastal systems [1], [39]–[41], and the importance of both groups as a source of food for macrobenthos [41]. The recycling of nutrients is another major feature of B-P coupling [42], but nutrient recycle terms are not included in the model to maintain simplicity. Our model excluded the state equation for suspension feeders in the Chesapeake Bay Eutrophication Model, and it combined macrobenthic suspension and deposit feeders into a single state equation. The benthic suspension feeder equation was not included in our model because its construction was based on large bivalve suspension feeders [28] that are generally rare in the lower Rappahannock River [43]. Based on the community composition of macrobenthos collected during the summers of 2007 and 2008 [36], a single governing equation for macrobenthos was assumed to be sufficient to model macrobenthic response to DO concentrations in the lower Rappahannock. Many of the macrobenthos in our samples demonstrate both suspension and deposit feeding traits (Table 1), and no definable difference in response to hypoxia was observed from either group in terms of a change in biomass [36]. The combination of these two groups also maintained our goal of keeping the model as simple as functionally possible.

Water quality variables (DIN, POC, and DO) were obtained from daily interpolations of Chesapeake Bay Water Quality Monitoring Program data from 1985 to 2001 [44], with the exception of photosynthetically active radiation (PAR) and water temperature. Daily PAR and water temperature were forced using equations derived by Wetzel and Neckles [45] for lower Chesapeake Bay.

### 2.4 Adaptations to the Original Model

Some specific changes were made to the original governing equations of the Chesapeake Bay Eutrophication Model (addressed below). The formulation that represented the response of zooplankton mortality to hypoxia (*Mz*) was altered; in our model, if DO concentration was less than 2 mg DO l^−1^ then:
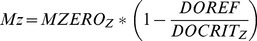
(4)in which:

*Mz*  =  hypoxic mortality of zooplankton group *Z* (d^−1^)

*MZEROz*  =  mortality at zero dissolved oxygen concentration (d^−1^)

*DOREF*  =  dissolved oxygen concentration when DO < *DOCRIT_z_*, otherwise 2 (mg DO l^−1^)

*DOCRITz*  =  threshold below which dissolved-oxygen-induced mortality occurs, this value equals 2 (mg DO l^−1^).

In the original equation *DOCRITz* was always 2 mg O_2_ l^−1^, and *DOREF* was the dissolved oxygen concentration when DO < *DOCRITz*, otherwise it was zero. However, during model simulation this resulted in a linear increase in modeled zooplankton population through time, therefore, the formulation was amended to equation 4, where the *DOREF* was the dissolved oxygen concentration when DO < *DOCRITz*, otherwise it was 2 mg DO l^−1^.

The parent Chesapeake Bay Eutrophication model simulates three fractions of sediment organic carbon, a labile, semi-labile, and refractory pool. In the original version of the macrobenthos model, the following portion of Eq. 3, 
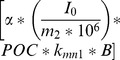
, was computed twice, once for the labile and once for the semi-labile carbon pool. Since our model was not coupled to a larger eutrophication model, in the interest of maintaining simplicity we computed this term in Eq. 3 once using total sediment POC from field measurements.

### 2.5 Rappahannock Function Relating Biomass to Hypoxia

In the original Eutrophication model, the impact of DO concentration on macrobenthos respiration (*r*), ingestion (*I_0_*), and mortality (*m*) was represented by the sigmoid equation *Z*, where:
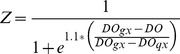
(5)where:

DO*_gx_*  =  DO at which macrobenthos function is 50% of maximum.

DO*_qx_*  =  DO at which macrobenthos function is 25% of maximum.

**Figure 1:**
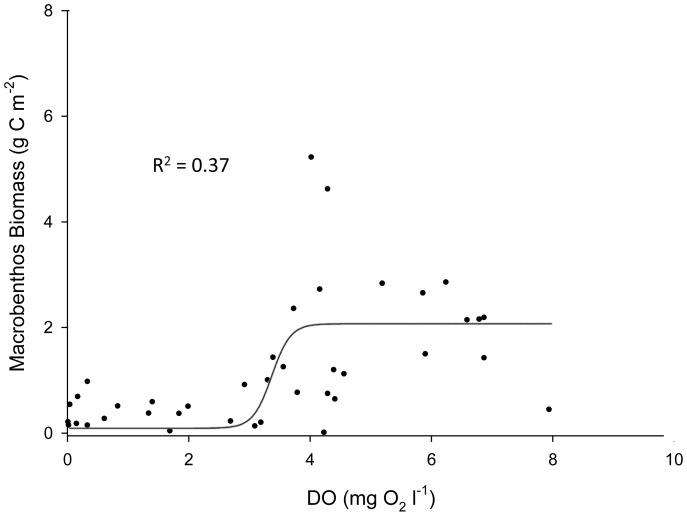
Relationship between DO concentration and macrobenthos biomass. Comparison of macrobenthos biomass and DO concentration from the four Rappahannock River sites monitored bi-weekly from May to October. Trendline is a sigmoid curve, where equation 
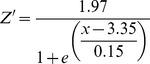
.

In the case of hypoxia-induced mortality, *Z* is then used in Eq. 5 above. The sigmoid equation that represents *Z* was not supported by any data. Our analysis of Chesapeake Bay field data and results from Seitz et al. [24] suggests a sigmoid relationship between DO and macrobenthic biomass, as mortality occurs below rather sharply defined oxygen concentrations. Therefore, a sigmoid function was empirically constrained using macrobenthos data collected from the Rappahannock River during the summers of 2007 and 2008 (Fig. 1), and a function (*Z'*) was fit to the data to model the impact of DO concentration on macrobenthicbiomass:
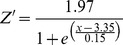
(6)


It is important to note that these relationships are quantitative representations of the actual various underlying causal factors between DO and macrobenthic biomass. In our model, *Z* is still used to model the impact of DO concentration on *r* and *I_0_* (see [38]), but *Z'* is used to more accurately model the impact of DO on macrobenthic biomass, replacing *Z* in Eq. 5 and providing an empirically constrained method for estimating hypoxia-induced mortality. Equation 6 was normalized (0 to 1, dimensionless) by replacing the numerator with 1, such that Equation 7 was the version applied in our model:
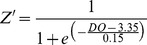
(7)where:

DO  =  dissolved oxygen concentration (mg l^−1^).

### 2.6 Model Verification and Simulation Analysis

A single model run encompassed a period of 365 days with a time step of one calculation per day. Model output for the three governing equations were verified using Chesapeake Bay Benthic, Water Quality, and Plankton Monitoring Program data from stations LE3.2, LE3.4, and LE3.6 which are located approximately 25 km, 19 km, and 2 km from the mouth of the Rappahannock River, respectively. While the stations used for verification differ spatially, the physical dynamics over this relatively small spatial scale of the lower Rappahannock River are not significantly different [44]. The macrobenthic output was verified with data from stations LE3.2 and LE3.4, and data from station LE3.6 were used to verify the phytoplankton and zooplankton equations; three stations were used due to data availability. Model output was visually compared to observations to determine validity [46], and by assessment of mean percent error (MPE) and root mean square deviation (RMSD) [32].

Predicted phytoplankton biomass was verified against the mean annual cycle of chlorophyll-a concentrations computed using Chesapeake Bay Program data from 1985 to 2001. To reflect mean water column conditions, this annual cycle was based on averages of surface and bottom concentrations; differences between the surface and bottom were usually less than 10 mg m^−3^. Predicted zooplankton biomass was verified using computed average annual cycles of combined micro- and mesozooplankton biomass using Chesapeake Bay Program data from 1985 to 2001. Depth-integrated zooplankton counts from the Plankton Monitoring Program were converted to biomass using species-specific carbon contents [47]. Predicted macrobenthic biomass was verified using Bay Program data from 1992, using site LE 3.4 for verification under normoxia and site LE3.2 for verification under hypoxia. Year 1992 was chosen at random from years 1985–2001. The approach of using a randomly selected year for macrobenthos verification instead of averaged annual cycles was due to the stochastic nature of benthic data collection through time; data were collected at variable dates each year by the Chesapeake Bay Program. Following verification, sensitivity analyses were conducted for the phytoplankton, zooplankton, and macrobenthic state variables by adjusting selected parameters that directly impacted growth or loss (i.e. consumption or predation parameters). Maximum photosynthetic rate (*P^b^m*), phytoplankton settling velocity (*Wa*), and predation rate on algae (*Phtl*) were tested for the phytoplankton state variable; predator biomass and clearance rate (*PHTlz*) for the zooplankton state variable; and assimilation efficiency for carbon (α) and ingestion limitation (*K_1_*) for the macrobenthic state variable. All parameters tested in sensitivity analysis were adjusted at an increment of ±20% and the relative percent difference from the standard run was calculated for each. Parameters with percentage differences greater than 10% were deemed to be sensitive parameters [48].

**Figure 2:**
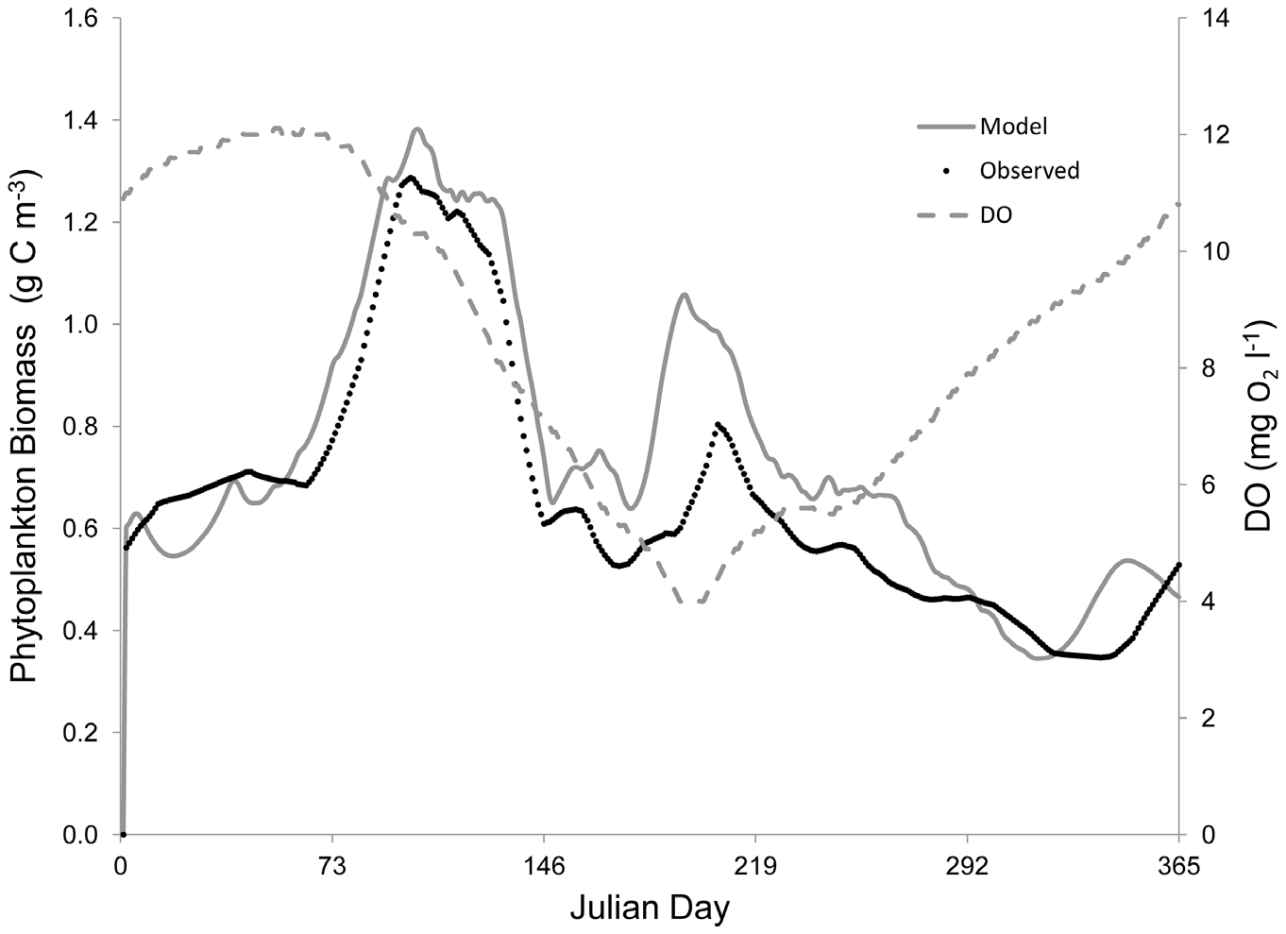
Verification of the phytoplankton state variable. Left y-axis: the gray solid line represents modeled phytoplankton biomass and the black dots denote mean observed phytoplankton biomass from site LE3.6 of the Chesapeake Bay Water Quality Monitoring Program from 1985–2001. Right y-axis: the grey dashed line indicates the dissolved oxygen concentration. Modeled phytoplankton biomass matched the trend and magnitude of observed phytoplankton biomass in 1992.

**Figure 3:**
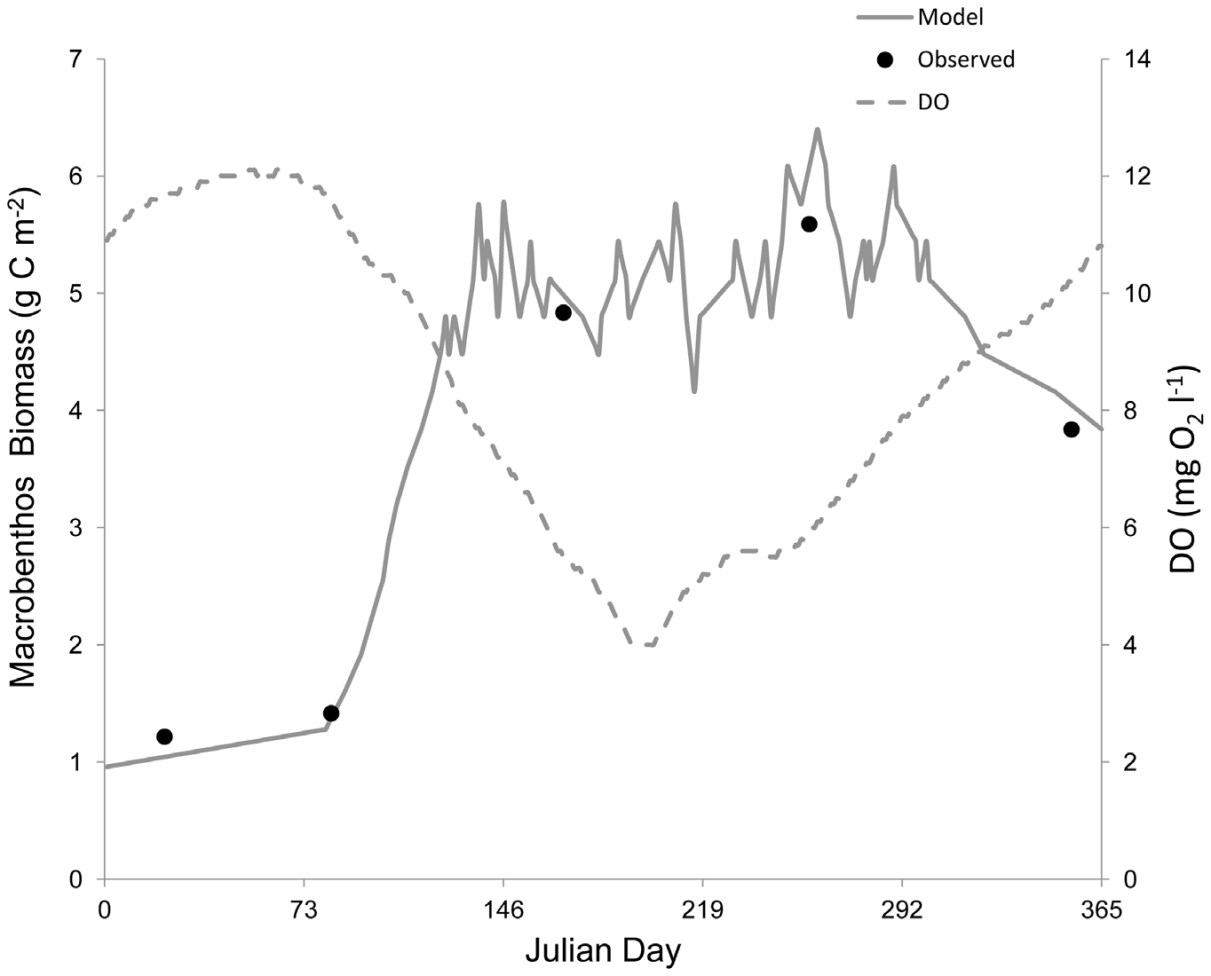
Verification of the macrobenthos state variable during normoxia. Left y-axis: the gray solid line represents modeled macrobenthos biomass and the black dots denote observed macrobenthos biomass from site LE3.4 of the Chesapeake Bay Benthic Monitoring Program in 1992. Right y-axis: the grey dashed line indicates the dissolved oxygen concentration. Modeled macrobenthos biomass matched the trends and magnitude of observed macrobenthos biomass in 1992.

**Figure 4:**
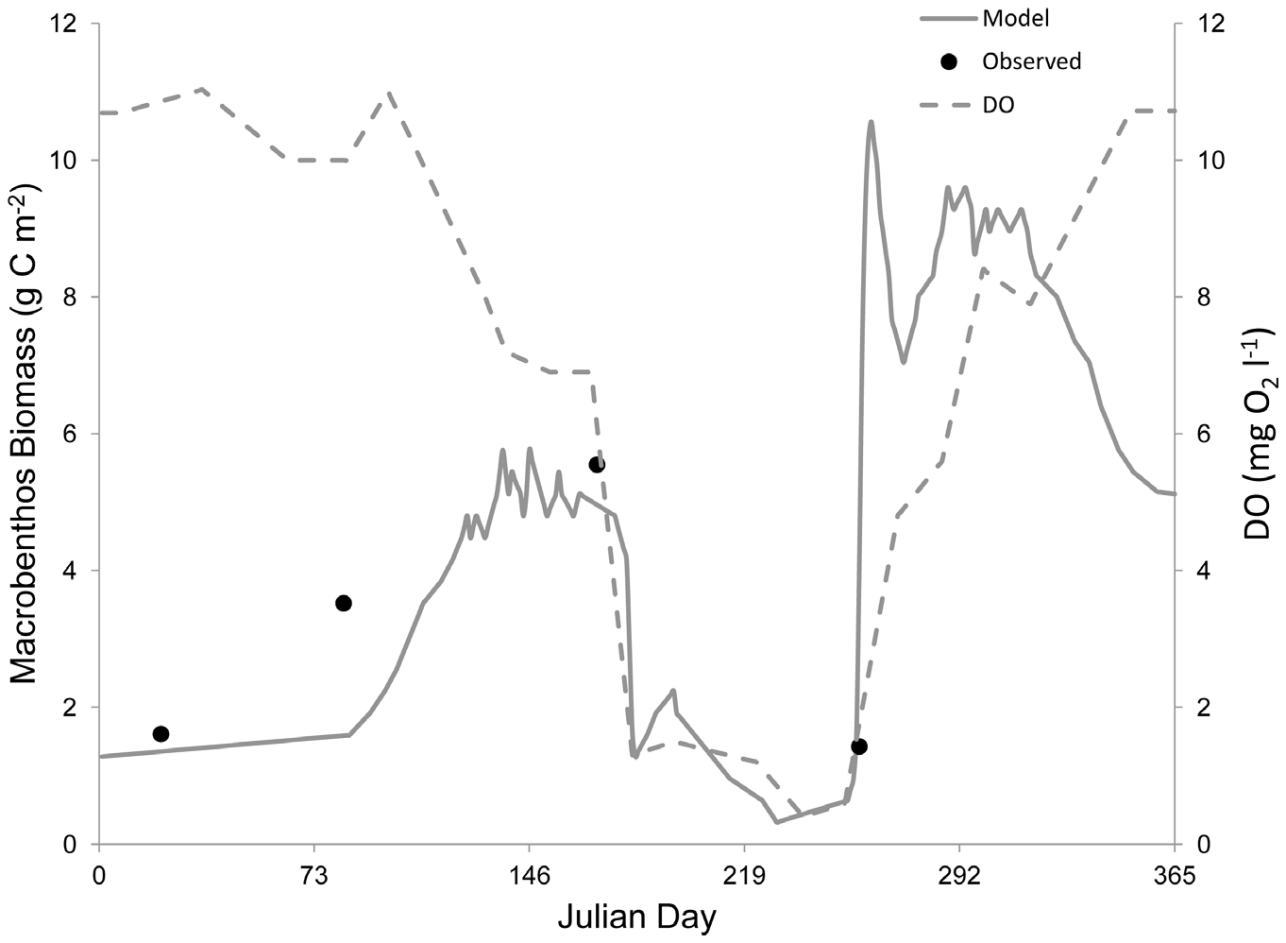
Verification of the zooplankton state variable. Left y-axis: the gray solid line represents modeled zooplankton biomass and the black dots denote mean observed zooplankton biomass from site LE3.6 of the Chesapeake Bay Water Quality Monitoring Program from 1985–2001. Right y-axis: the grey dashed line indicates the dissolved oxygen concentration. Modeled zooplankton biomass approximated the observed annual cycle and matched the magnitude of observed zooplankton biomass in 1992.

A set of simulations analyses were then conducted, adjusting DO concentration to model the effect of the severity and duration of hypoxia on the three modeled state equations (Table 2). DO concentrations were gradually adjusted during a period of 3 days to avoid artificial rapid changes in the model. Simulations S1 to S3 focused on the sustained duration of hypoxia. S4 simulated the development of hypoxia during the neap-spring tidal cycle with DO cycling for 14 days. Verified model output from the state equations (i.e. the base model results during normoxia after model verification and sensitivity analyses; Fig. 2, 3, and 4) was used as a baseline for comparison against simulations S1 to S4. This was conduct through visual observation and comparison of the means and standard deviations between the verified model output and the simulations (e.g. phytoplankton, macrobenthos, and zooplankton output in S1 was compared to the models in Fig. 2, 3, and 4, respectively). Simulations S5 to S9 modeled the severity of hypoxia from 0.0 to 2.0 mg O_2_ l^−1^ with a 0.5 mg O_2_ l^−1^ step and constant duration of 60 days. Comparison of means and standard deviations were used to justify “significant” differences. DO concentration was forced for each model simulation, with consideration of historical data of DO dynamics in the Rappahannock River [44].

**Table 2:** Model simulations.

Simulations	Hypoxia Duration (d)	Julian Day	Ordinal Date	DO (mg O_2_ l^−1^)
S1	120	148–267	May 28– Sept 24	0.5
S2	60	178–237	Jun 26– Aug 26	0.5
S3	30	191–222	Jul 11– Aug 11	0.5
S4	14 d intervals	162–176, 190–204, 218–232, 246–260	Jun 11–25, Jul 9–23, Aug 6–20, Sept 3–17	0.5
S5	60	178–237	Jun 26– Aug 26	2.0
S6	60	178–237	Jun 26– Aug 26	1.5
S7	60	178–237	Jun 26– Aug 26	1.0
S8	60	178–237	Jun 26– Aug 26	0.5
S9	60	178–237	Jun 26– Aug 26	0.0

Ecosystem model simulations with varying hypoxic duration and severity. S1 to S4 (Figures 6 and 7) modeled hypoxic duration at a constant concentration of 0.5 mg O_2_ l^−1^; S4 modeled intermittent hypoxia (hypoxia occurring every 14 days on a neap/spring tidal cycle); S5 to S9 modeled the affect of hypoxic severity at a constant duration of 60 days.

## Results

**Figure 5:**
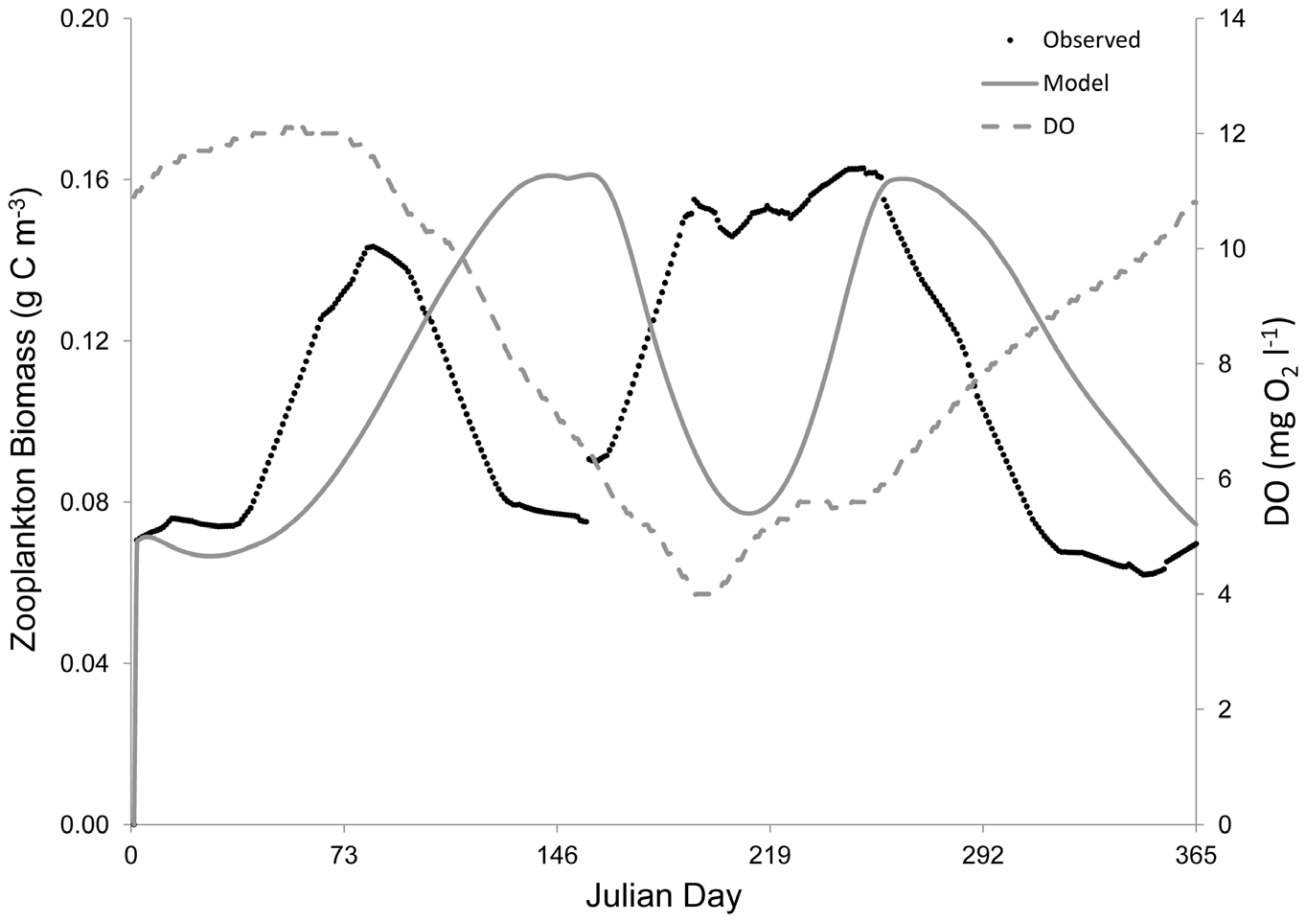
Verification of the macrobenthos state variable during hypoxia. Left y-axis: the gray solid line represents modeled macrobenthos biomass and the black dots denote observed macrobenthos biomass from site LE3.2 of the Chesapeake Bay Benthic Monitoring Program in 1992. Right y-axis: the grey dashed line indicates the dissolved oxygen concentration. Modeled macrobenthos biomass matched the trends and magnitude of observed macrobenthos biomass in 1992.

Modeled phytoplankton biomass (*P*) matched the general trends in observed biomass in the lower Rappahannock River, with blooms and declines consistent with the data from 1992 (Fig. 2). The model also reproduced the approximate magnitude of phytoplankton biomass with MPE of 13.3% and RMSD of 0.18 g C m^−3^. Modeled macrobenthic biomass (*B*) also matched the patterns in observed biomass in 1992 at both a normoxic site (Fig. 3) and a hypoxic site (Fig. 5). The model accurately portrayed the temporal dynamics in macrobenthic biomass with respect to DO concentration, as well as the magnitude of biomass with MPE of 12.1% and RMSD of 0.56 during normoxia and MPE of 15.7% and RMSD of 0.22 g C m^−2^ during hypoxia. Modeled zooplankton biomass matched the magnitude of observed biomass in 1992, but did not follow the trends in the observed data (Fig. 4), with MPE of 4.5% and RMSD of 0.38 g C m^−2^. Modeled zooplankton dynamics appeared to be delayed when compared to observed increases and decreases in zooplankton biomass. This discrepancy may be due to the combination of micro- and mesozooplankton into a single state variable. Additionally, there were noted calibration difficulties for zooplankton in the parent eutrophication model [49], which may have extended into our adaptation of that model.

Discrepancies between modeled and observed zooplankton biomass could be expected to have detrimental effects on modeled phytoplankton biomass, given the significant influence of zooplankton predation rate on algae (see Table 3 below). However, modeled phytoplankton biomass successfully reproduced the observations (Fig. 2). Additionally, phytoplankton biomass in the lower Rappahannock River is 3.3–14.3 times greater (mean = 6.5) than zooplankton biomass (Fig. 2 and 4), which indicates that phytoplankton will have the dominant effect on modeled macrobenthic biomass. The successful simulation of phytoplankton biomass, simulation of zooplankton biomass in the correct range (Fig. 4), the overall much greater biomass of phytoplankton compared to zooplankton, and the accurate simulation of macrobenthic biomass under both normoxia (Fig. 3) and hypoxia (Fig. 5), confirm the ability of the model to predict the dynamics of macrobenthic biomass in response to hypoxia in further simulation analyses.

**Table 3:** Sensitivity analysis.

State variable	Parameter	−20%	+20%	Average RMS	% Diff -20%	% Diff +20%
Phytoplankton	*P^b^m*	0.032	0.041	0.037	12.7*	11.8*
	*Wa*	0.041	0.032	0.037	11.5*	12.2*
	*Phtl*	0.044	0.030	0.037	18.8*	18.6*
Zooplankton	*PHTlz*	0.001	0.003	0.002	11.8*	13.1*
Macrobenthos	*α*	0.118	0.369	0.192	38.8*	91.8*
	*K_1_*	0.191	0.194	0.192	0.8	0.5

Results of sensitivity analysis for phytoplankton, zooplankton, and macrobenthic state variables. The root mean square (RMS) was the average variance in the base state equation over all time steps of a single year (n = 365). RMS values are shown for ±20% variation for each state variable by parameter. The model was deemed to be sensitive when % difference exceeded 10% (Ragone-Calvo et al. 2001). Parameters: Maximum photosynthetic rate (*P^b^m*), phytoplankton settling velocity (*Wa*), predation rate on algae (*Phtl*), predator biomass and clearance rate (*PHTlz*), assimilation efficiency for carbon (α), and ingestion limitation (*K_1_*).

**Denotes model sensitivity.**

Sensitivity analyses were conducted on model parameters for each state variable (Table 3). The model was sensitive to a majority of tested parameters, with the phytoplankton state variable sensitive to all tested parameters. The zooplankton state variable was found to be sensitive to an increase and decrease in predator biomass and clearance rate. The macrobenthic state variable was sensitive to assimilation efficiency for carbon and insensitive to ingestion limitation. The sensitivity of these parameters is a problem in the parent model that we did not attempt to address. The goal of this manuscript was not to resolve nuances in the original model, but to take a simple approach and constrain the effect of DO concentration on macrobenthic biomass, within the context of the more complex Chesapeake Bay Eutrophication Model.

**Figure 6:**
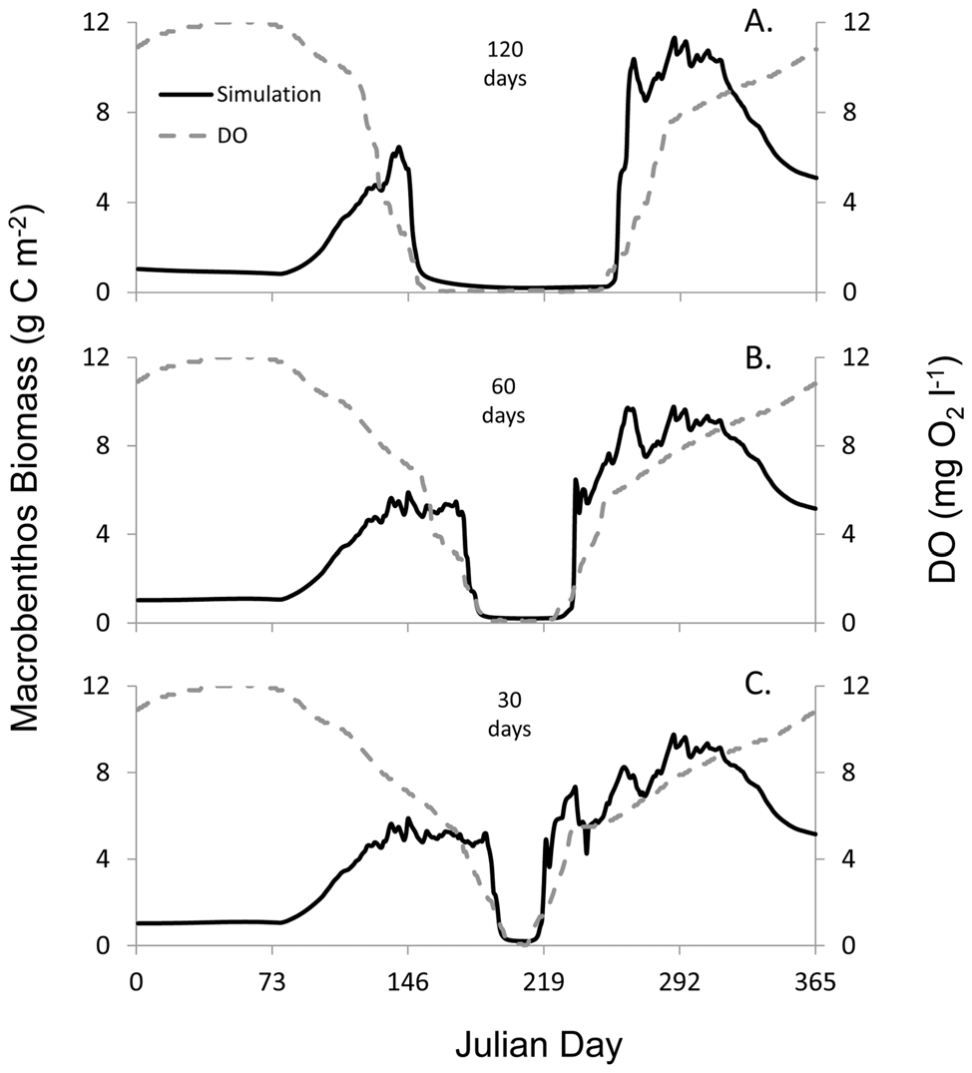
Simulations of hypoxic duration and macrobenthic response. Simulated macrobenthos biomass (*B*) under hypoxic durations of (A) 120, (B) 60, and (C) 30 days; S1, S2, and S3, respectively. Left y-axis: the gray solid line represents modeled macrobenthos biomass and the black dots denote observed macrobenthos biomass from site LE3.4 of the Chesapeake Bay Benthic Monitoring Program in 1992. Right y-axis: the grey dashed line indicates the dissolved oxygen concentration. *B* was influenced by the duration of hypoxia.

Simulations were run assessing the impact of hypoxic duration on phytoplankton, zooplankton, and macrobenthic biomass. Macrobenthic biomass began to decrease as scenario S1, which simulated a hypoxic duration of 120 days, approached hypoxia (Fig. 6A). At the start of hypoxia in S1 the steady decrease in macrobenthic biomass accelerated to an immediate collapse in biomass that lasted the duration of the hypoxic event, with a temporal trend in macrobenthic biomass different from that modeled under normoxic conditions. Macrobenthic biomass began to respond and increase before DO concentrations in S1 became normoxic. A few days after hypoxia ended, macrobenthic biomass had increased to above pre-hypoxia levels. Similar trends were observed in S2 and S3 involving hypoxic durations of 60 and 30 days (Fig. 6B and C), with the length of the collapse the main difference in macrobenthic biomass. Simulations with shorter durations of hypoxia resulted in less time with macrobenthic biomass near 0 g C m^−2^. Macrobenthic biomass of S2, with a mean of 4.1 g C m^−2^ (SD = 3.1), and S3, with a mean of 4.4 g C m^−2^ (SD = 2.8), were different than the macrobenthic biomass model under normoxia, with a mean of 3.5 g C m^−2^ (SD = 1.5).

**Figure 7:**
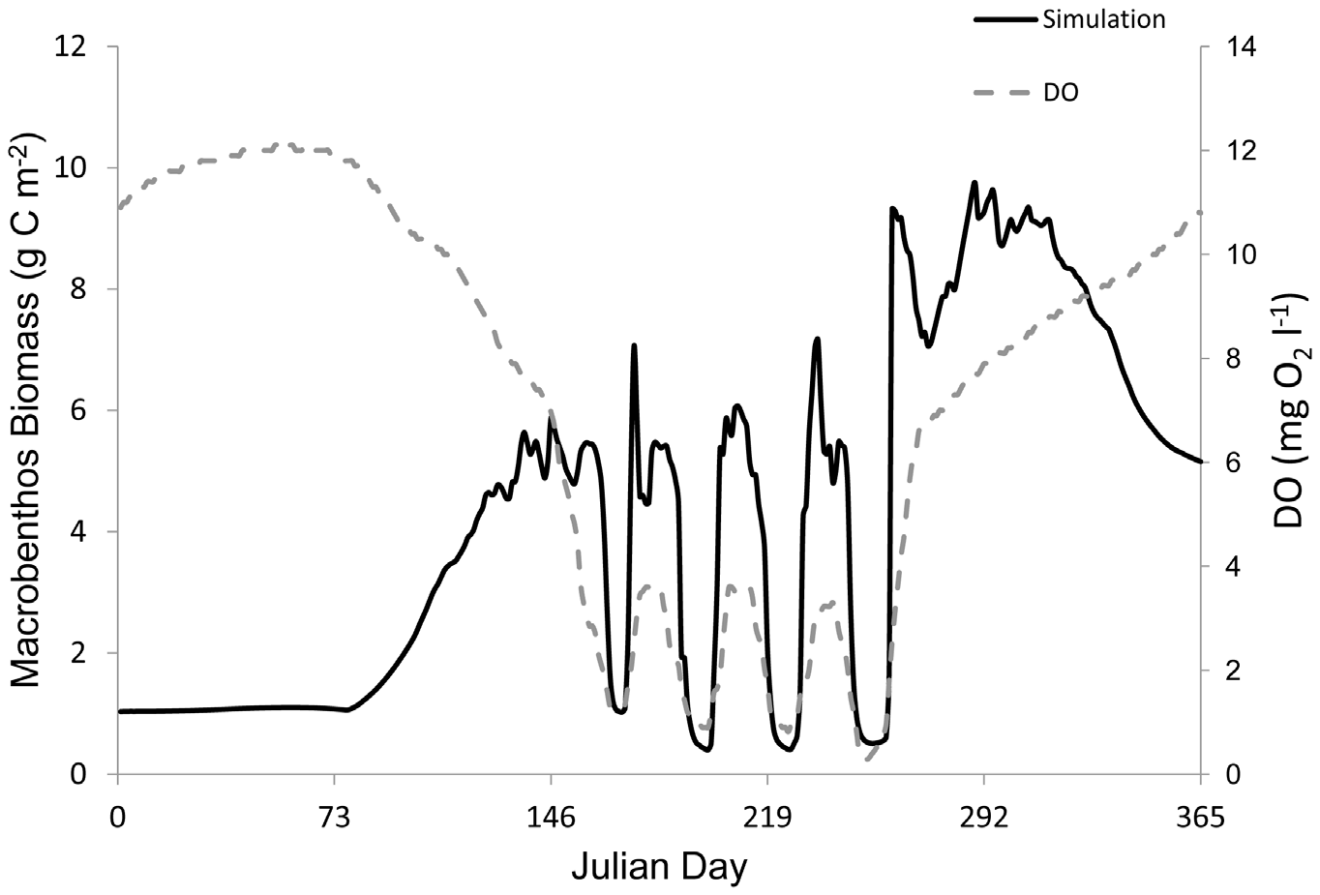
Simulations of intermittent hypoxic duration and macrobenthic response. Simulated macrobenthos biomass (*B*) under intermittent hypoxia (14 days hypoxic and 14 days normoxic) representing effect of neap/spring tide cycle. Left y-axis: the gray solid line represents modeled macrobenthos biomass and the black dots denote observed macrobenthos biomass from site LE3.4 of the Chesapeake Bay Benthic Monitoring Program in 1992. Right y-axis: the grey dashed line indicates the dissolved oxygen concentration on the right y-axis. *B* responded to modeled intermittent hypoxia.

In simulation S4, with intermittent hypoxia, macrobenthic biomass decreased at the onset of hypoxia and remained near zero for the duration of hypoxia (Fig. 7). During the 14-day intervals when DO concentration was normoxic, macrobenthic biomass began to increase but decreased back near zero with the onset of hypoxia. Macrobenthic biomass fluctuated through this pattern throughout the hypoxic simulation. A few days after hypoxia abated permanently, macrobenthic biomass began to increase to biomass levels greater than those observed pre-hypoxia.

**Figure 8:**
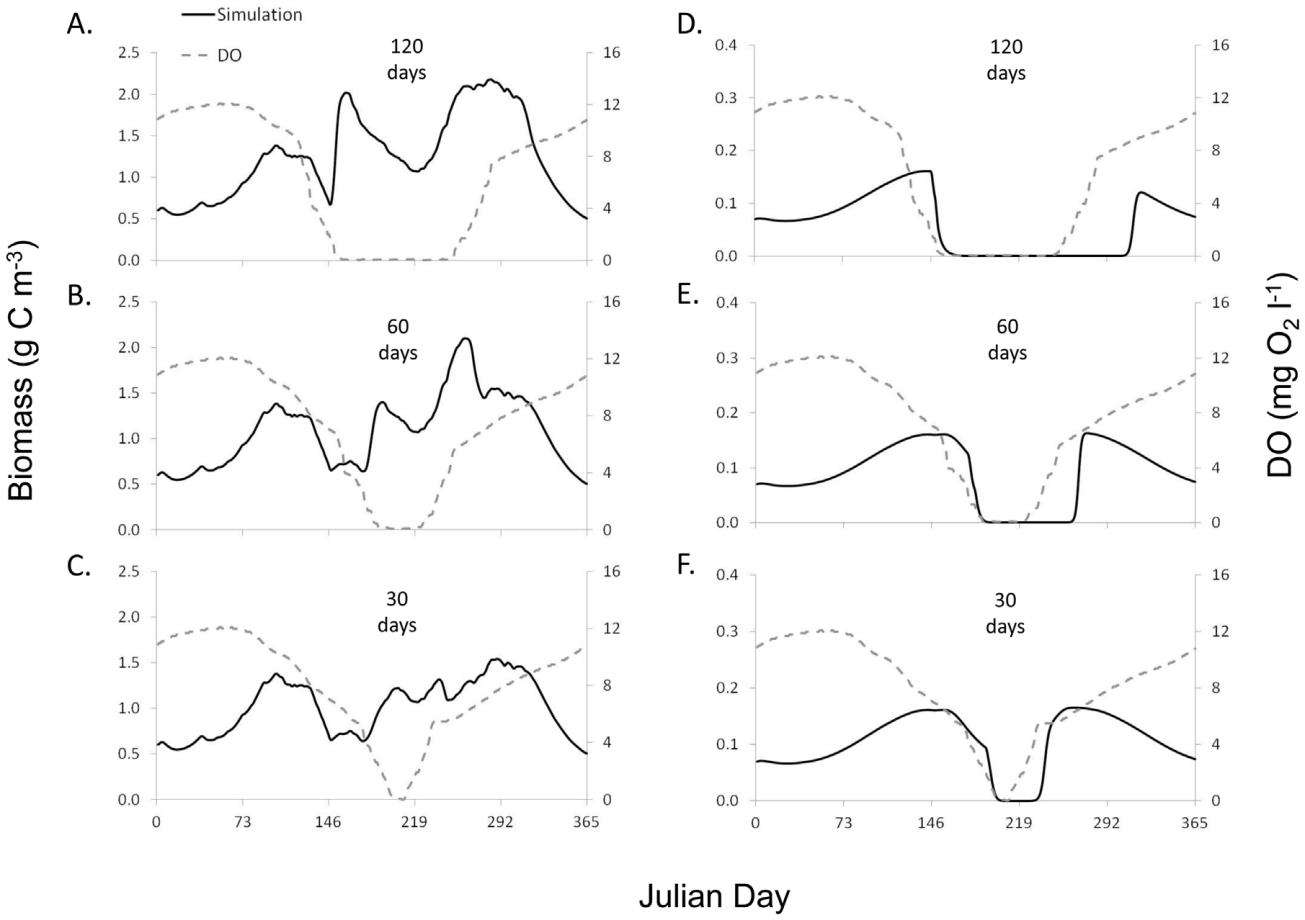
Simulations of hypoxic duration and phyto- and zooplankton response. Simulated phytoplankton biomass under hypoxic durations of (A) 120, (B) 60, and (C) 30 day, and zooplankton biomass under hypoxic durations of (D) 120, (E) 60, and (F) 30 days. Left y-axis: the gray solid line represents modeled biomass and the black dots denote mean observed biomass from site LE3.6 of the Chesapeake Bay Water Quality Monitoring Program from 1985–2001. Right y-axis: the grey dashed line indicates the dissolved oxygen concentration on the right y-axis. Modeled phytoplankton and zooplankton biomass responded inversely to hypoxic simulations.

Modeled phyto- and zooplankton biomass responded oppositely during hypoxic simulations (Fig. 8). As DO concentrations began to decline, phytoplankton biomass initially decreased, however, with the onset of hypoxia, phytoplankton biomass increased. The length of increased phytoplankton biomass was dependent on the duration of hypoxia, with a longer duration of hypoxia resulting in higher overall phytoplankton biomass, and to some extent an even greater magnitude of phytoplankton biomass. Hypoxia had the reverse effect on zooplankton biomass. As DO concentration decreased to hypoxic levels, zooplankton biomass initially increased and then declined to near 0 g C m^−3^. The length of time that zooplankton biomass stayed near 0 g C m^−3^ was dependent on the duration of hypoxia; lengthy durations of hypoxia coincided with longer durations of reduced zooplankton biomass. Modeled zooplankton biomass did not respond to increased DO concentration until days to weeks after hypoxia ended.

The effect of hypoxic severity on macrobenthic biomass was tested by adjusting DO concentration between 2.0 and 0.0 mg O_2_ l^−1^ in increments of 0.5 mg O_2_ l^−1^. Macrobenthic biomass was not substantially different between simulations when compared during the full year (Table 4). The mean biomass of these four simulations was approximately equal, with large standard deviations. However, these simulations had similar macrobenthic biomass during normoxia, as no parameters were changed; macrobenthic biomass did not differ between simulations until DO concentrations became hypoxic. Therefore, simulations S5 to S9 were analyzed starting at the onset of hypoxia on day 178 through the end of hypoxia on day 237 (Table 5). S5 had appreciably higher macrobenthic biomass than S6 to S9. Mean biomass in S5, which depicted 60 days of hypoxia at 2.0 mg O_2_ l^−1^, was >5 times higher than simulations that modeled hypoxia at DO concentrations of 0.0–1.0 mg O_2_ l^−1^, and 3 times higher than S6. In S6 when the DO was 1.5 mg O_2_ l^−1^ macrobenthic biomass was lower than S5 and >2 times higher than S7 to S9. There were negligible differences in biomass at DO concentrations of 1.0 mg O_2_ l^−1^ and lower. The mean biomass in S7, S8, and S9 was approximately similar with large standard deviations.

**Table 4:** Response of macrobenthic biomass to hypoxic severity (365 d).

Simulation	DO (mg O_2_ l^−1^)	Julian Day	Mean Biomass (g)
S5	2.0	1–365	3.20 (1.84)
S6	1.5	1–365	3.04 (2.07)
S7	1.0	1–365	2.88 (2.07)
S8	0.5	1–365	2.88 (2.07)
S9	0.0	1–365	2.88 (2.07)

Comparison of macrobenthos biomass to hypoxic severity over a full year. Macrobenthos biomass was not measurably different between simulations over a full year.

**Table 5:** Response of macrobenthic biomass to hypoxic severity (60 days).

Simulation	DO (mg O_2_ l^−1^)	Julian Day	Mean Biomass (g)
S5	2.0	178–237	4.07 (0.46)
S6	1.5	178–237	1.77 (0.69)
S7	1.0	178–237	0.76 (1.15)
S8	0.5	178–237	0.62 (1.15)
S9	0.0	178–237	0.62 (1.15)

Comparison of macrobenthos biomass to hypoxic severity over a partial year, covering the 60 day time-frame of simulated hypoxia. Macrobenthos biomass was measurably variable during the 60 day assessment. Mean biomass is shown with ±1 SD in parentheses.

## Discussion

The sigmoid function (*Z'*) applied to this ecosystem model resulted in clear changes in simulated macrobenthic biomass (*B*) during various hypoxic scenarios. In our model, hypoxic duration resulted in prolonged reductions of macrobenthic biomass relative to the length of hypoxia, with the model suggesting near defaunation (macrobenthic biomass equal to 0 g C m^−2^) during the 120, 60, and 30 day hypoxic scenarios (simulations 1, 2, and 3, Fig. 6) at a DO concentration of 0.5 mg O_2_ l^−1^. This is in good agreement with laboratory and field studies that show duration and severity of hypoxia to impact benthic communities (see [11], [26]). Periods of prolonged hypoxia have been observed previously in Chesapeake Bay and elsewhere [8], [50]. The deep trough of the mainstem Bay experiences sustained seasonal hypoxia year after year [15]. While some species have documented resistance to hypoxia [7], during extended periods of hypoxic exposure (∼40 days) even the most tolerant of species experience total mortality [51].

After DO levels in our model returned to normoxia, macrobenthic biomass recovered greater than pre-hypoxic levels. This was unexpected as conditions in Chesapeake Bay that stimulate ecological production in the pre-hypoxic spring differ in the post-hypoxic fall [8]. Increased nutrient run-off from the spring freshet promotes plankton production; the particulate organic matter from these blooms eventually settles to the bottom promoting benthic growth [52]. Large plankton blooms seen in the spring are noticeably absent in the fall, and with less primary production one would expect the rate of recovery of macrobenthic biomass to be less in the fall than in the spring. However, data from the continuously monitored sites used to derive *Z'* indicated macrobenthic production can increase to pre-hypoxic levels a few weeks post hypoxia, suggesting the macrobenthic biomass increases we observed post-hypoxia may be reasonable [36]. A decrease in predation pressure may also have contributed to the observed increase in macrobenthic biomass. In an ecological context, decreases in temperature during the fall and winter months post-hypoxia lower metabolic demand, and thus the need of predators to obtain food [53]. In our model this was accounted for through Arrhenius temperature dependencies in the predation formulation, which is a simple but accurate formula for the temperature dependence of reaction rates [54]. This however, suggests that during summer hypoxia, the loss of macrobenthic biomass and its impacts to higher consumers such as epibenthic predators and demersal fish is not compensated for by the increase in macrobenthic biomass post-hypoxia due to a change in metabolic demand and activity.

Modeled intermittent hypoxia (S4, Fig. 7) resulted in reduced macrobenthic biomass during hypoxic periods but some recovery during normoxia. This cycling continued throughout the intermittent series of hypoxic events. Given the severity at which the DO concentration was set for this simulation (0.5 mg O_2_ l^−1^), it is not surprising that macrobenthic biomass decreased to the observed level. Neubauer [55] found *in situ* decreases in instantaneous macrobenthic production coincided with a hypoxic event, however observed no cyclical pattern between macrobenthic production and hypoxia. Mean DO concentration during Neubauer's [55] hypoxic event were ≈2.1–2.5 mg O_2_ l^−1^, which would be considered mild hypoxia for macrobenthos [56]. Further, Neubauer [55] experienced large recruitment events for some macrobenthic species during hypoxia at the time of his study. In contrast, a cyclical pattern between hypoxia and macrobenthic production was observed in data from the continuously monitored sites used to derive *Z'*. DO concentrations at our *in situ* sites were as low as ∼0.02 mg O_2_ l^−1^ on two separate occasions, and the similarities in hypoxic severity between our model and our *in situ* observations may account for analogous results [36].

As with simulations S1, S2, and S3, the growth of macrobenthic biomass during simulation S4 was very rapid with the return of normoxia, an observation that was also documented *in situ* [36]. A number of the dominant macrobenthic species in the lower Rappahannock spawn and recruit throughout the summer [57]–[63]. The larvae of these species are found distributed throughout the water column, and this pattern does not change in response to low DO [64]. When favorable conditions return, the planktonic larvae of these benthic species are available to settle in an organic-rich environment with few competitors, which may explain why quick benthic recruitment post-hypoxia is observed. Modeled macrobenthic biomass also began recovering ∼2–3 days before hypoxia abated, which represents the time frame that DO was increasing from 0.5 to 2.0 mg O_2_ l^−1^, indicating a sensitivity to hypoxic severity. Macrobenthos have been previously documented to recruit before the DO concentration is above 2.0 mg O_2_ l^−1^, although this low level of DO does delay settlement of some hypoxia sensitive taxa [64]. In our intermittent simulation, once macrobenthic biomass reached a level where it appeared sustainable, hypoxia returned and biomass was reduced to near zero again.

Hypoxic severity had a measureable impact on macrobenthic biomass with higher biomass in simulations with less severe hypoxia. Studies have shown the severity of hypoxia to affect the response of benthic communities; the more severe the hypoxia, the greater the impact on the benthos, directly and indirectly [7]. Directly, benthic species vary in their tolerances to low DO concentrations [11], and as the severity of hypoxia increases towards anoxia, sensitive species die-off, usually due to asphyxiation, decreasing the diversity of the affected area and overall biomass [7]. Additionally, the toxic compound hydrogen sulfide is present in severely hypoxic sediments, and has been suggested as a mechanism contributing to macrobenthic mortality during hypoxia [65]. Indirectly, DO concentrations can positively and negatively affect benthic predation; Nestlerode and Diaz [66] showed that benthos may actually have a refuge from predation under mild hypoxic conditions, and Brante and Hughes [67] demonstrated that hypoxia reduced *Carcinus maenas* predation on mussels. If macrobenthos are able to avoid mortality via asphyxiation, such actions during prolonged hypoxic events could also indirectly lead to starvation [7].

During model simulations, there was no increase in macrobenthic biomass as DO concentrations declined towards hypoxia. In our model *β*:
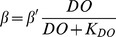
(8)in which: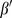  =  the predation rate before considering hypoxic effects*K_DO_*  =  predation DO half-saturation.

accounts for the predation rate on macrobenthos and denotes predation rate as a function of temperature and DO concentration, where predation increases with increasing DO. During mild hypoxia predators may not effectively prey upon benthos, and hypoxia tolerant benthos would survive and maintain their biomass. The model excludes the effect of increasing predation during hypoxia, an artifact of the parent model. Seitz et al. [68] and Long and Seitz [21] showed that epibenthic predators and demersal fish can at times capitalize on stressed benthos during mild hypoxic events. As oxygen concentrations become lethal, stressed macrobenthos extend their appendages and bodies out of the sediment in an attempt to escape severely hypoxic conditions below the sediment-water interface [56]. Opportunistic mobile predators have been shown to re-enter hypoxic areas and prey on exposed macrobenthos during mild hypoxia [69], [70].

Zooplankton biomass (*M*) was negatively impacted by hypoxia directly, causing zooplankton biomass to be drastically reduced. Marcus et al. [71] considered the effect of reduced DO concentration on the survival and population dynamics of zooplankton, demonstrating the deleterious effect hypoxia has on zooplankton population and community dynamics. In our model, phytoplankton biomass was sensitive to predation by zooplankton, with a 20% increase and 20% decrease in algal predation resulting in an 18% difference in phytoplankton biomass (Table 3). As a result, phytoplankton biomass (*P*) in our model was indirectly positively influenced by the onset of hypoxia, due to the release of phytoplankton from zooplankton grazing pressure.

## Conclusions

Macrobenthos data from the lower Rappahannock River were used to derive *Z'*, a sigmoid relationship, to model the effect of DO concentration on macrobenthic biomass (*B*). *Z'* was then used to empirically constrain a formulation for hypoxia-induced mortality in a biomass-based ecosystem model and used to assess the impact of hypoxia on macrobenthic biomass, while including the important biological interactions that occur through benthic-pelagic coupling. *Z'* is a useful tool in that it can be applied to existing models to simulate the impact of hypoxia on the macrobenthos, and the methods used to derive *Z'* can be applied to other systems to develop site specific and species specific parameterizations of *Z'*. Further, in terms of application, the simplicity of our model makes it easy to implement, interpret, and update, while at the same time the approach developed here can be readily incorporated into more complex models.

From our modeling efforts we found that the duration and severity of hypoxia negatively affected macrobenthic biomass; longer durations and greater hypoxic severity resulted in less biomass. Further, our model suggests that post hypoxia, macrobenthic biomass can return to pre-hypoxic levels, which implies a level of resiliency in the macrobenthic community to hypoxia. However, it is important to remember that the loss of macrobenthic biomass occurs at a critical time when energy demands of epibenthos and demersal fish, predators of macrobenthic organisms, are at their highest. It is unknown if the rebound in macrobenthic biomass observed in our model post-hypoxia would be enough to compensate for the loss in biomass during hypoxia.

The ecological importance of macrobenthos to estuarine systems underlies the significance in understanding processes that positively and negatively impact this group. The ability to accurately model the influence of low DO and strength of interactions between ecosystem components will improve our understanding of the impacts of hypoxia and provide a holistic view of a major anthropogenic stressor on ecosystem functioning.
