# Correction: Modeling the Effect of Hypoxia on Macrobenthos Production in the Lower Rappahannock River, Chesapeake Bay, USA

**DOI:** 10.1371/annotation/ad489273-be3a-42ca-acdc-b6ca5ec1abfb

**Published:** 2014-01-23

**Authors:** Samuel Kersey Sturdivant, Mark J. Brush, Robert J. Diaz

The graphs in figures 4 and 5 were incorrectly switched. The legend of figure 5 corresponds to the image labeled figure 4 and the legend of figure 4 corresponds to the image labeled figure 5. The references to these figures throughout the manuscript are correct.

